# The Book World of Medicine and Science

**Published:** 1901-10-19

**Authors:** 


### THE BOOK WORLD OF MEDICINE AND SCIENCE.

A Treatise on Plague: the Conditions for its Causation, Prevalence, Incidence, Immunity, Prevention and Treatment. By Major George S. Thomson, M.B., M.Ch., M.A.O., Roy.Univ.Irel., I.M.S.; and Dr. John Thomson, M.R.C.S.I., L.R.C.P.I. (London: Swan, Sonnenschein and Co. 1901. Price 7s. 6d. net.)

Incidentally this little book gives a good account of the phenomena of plague. This, however, is not the object with which the authors set out to write it, but rather to insist upon certain points in regard to its etiology and certain measures in its prevention. They grant at once that the microbe discovered by Kitasato is the essential cause of the disease; but they hold, and this is their great point, that the conditions favourable for its development, the conditions without which, in fact, it cannot spread, are those influences which devitalise the air, such as the crowding together of many unclean individuals in a small space, the absence of sanitary measures, and the deficiency of ventilation produced by narrow streets and badly constructed dwellings, and favoured by a hot, humid atmosphere, with only moderate winds or a complete calm. It is obvious enough that there is much to be said in favour of this theory. What strikes us is that it is not entirely novel. Indeed, if the authors had merely stated that by living in the insanitary conditions which they so fully describe a predisposing susceptibility to the disease is set up, we fancy that they would find themselves supported by nearly all careful observers. They seem, however, to go somewhat further and to attribute to a want of fresh air such a preponderating influence in the etiology of plague that all the rest sinks into insignificance. Taking, for example, the question of rats, they admit that plague is a disease of rats which is communicable to man, but they hold that the extermination of the rat is as futile a measure as it would be to attempt to exterminate the germ. "Both, if feasible, would practically abolish the disease; but human beings suffer because they live like rats, congregated in a filthy manner. When people have changed their habits and habitations, and live as civilised human beings should do, in clean, spacious, airy, and open houses, with ample cubic space for each occupant, and clean surroundings, the rats may freely invade their dwellings but no plague will ever come nigh them." This is a good sample of the general drift of the book. It sets out with great force the vast influence of environment in determining the fate of an infection, and in so doing it teaches a great lesson which applies not to plague alone. It is in fact the parable of the sower over again. We must think of the ground rather than of the seed. "The fever only becomes contagious by accumulation of people in ill-ventilated and dirty apartments. If disinfection is adopted as a substitute for thorough ventilation it is injurious; if only super-added it is superfluous."

Manual of Diseases of the Ear, including those of the Nose and Throat in Relation to the Ear. By Thomas Barr, M.D. Third edition, revised and partially re-written, with 236 illustrations. (Glasgow: James Maclehose and Sons. 1901. Price 12s. 6d. net.)

A third edition of this very useful manual having been called for Dr. Barr has taken the opportunity of bringing it up to date throughout, a process which has involved the re-writing of some portions of it. The outcome of his labours is a remarkably clear and complete account of the diseases of the ear. The principal changes in this edition are to be found in those chapters which deal with the results of purulent disease of the middle ear, and in the account of the operative measures which they require for their treatment. A good description is given of the various modern operations which have come into vogue during recent years, and among others will be found full details of the operation in two stages lately introduced by Ballance with the object of facilitating the healing of the cavities left after mastoid operations by means of the application of epithelial grafts. Much has been learned during late years in regard to the intracranial and vascular infective complications of purulent inflammation of the middle ear, and here we find a very good account of the whole problem. Both students and practitioners will, we are sure, find this a most straightforward and trustworthy guide to the management of diseases of the ear. Its diction is easy, its printing is good, and its illustrations are admirable. Our chief criticism would be in regard to the lack of definiteness as to antiseptic methods, especially those now so generally employed in the preparation of the ear for operation.

Some Retrospects and Prospects in Surgery. By Reginald Harrison, F.R.C.S. (John Bale, Sons and Daniellsson. London. 1901.

This pamphlet contains the substance of an address delivered at the Medical School of Cornell University, New York, on March 13th, 1901. The author deals with the subject of the surgery of the urinary organs, and gives a short account of the history of litholapaxy as instituted by Professor Bigelow. He next lays great stress on the importance of research in physiological chemistry in order to determine the substances which enter into, and the mode of formation of, urinary calculi, and quotes a most instructive case in which a pedunculated growth of prostate tissue converted the lower and posterior segment of the bladder into a "box." The retention of the urine led to the formation of two calculi as large as filberts. Mr. Harrison concluded his interesting address by a reference to the method of relieving kidney diseases depending upon tension by reni-puncture.

Lecture on Physiology for Teachers. By C. S. Sherrington, M.A., M.D.Camb., F.R.S. (Childhood Society for the Scientific Study of the Mental and Physical Conditions of Children.)

This interesting pamphlet contains many useful hints to teachers concerning the normal physiological functions and their bearing on the management of child life. The author draws attention to the necessity of attending to the teeth, to rest after meals, to the position of the blackboard and books in reading, and to physical exercise. The whole is argued on truly scientific physiological principles.

